# The lipid-lowering effects of Danhong and Huangqi injections: a meta-analysis of clinical controlled trials

**DOI:** 10.1186/s12944-018-0760-2

**Published:** 2018-05-10

**Authors:** Liuqin Yu, Chunyang Zhou, Zhi Luo, Wei Zeng, Feiya Lai, Gangjie Han, Yongyan Song

**Affiliations:** 1 Institute of Materia Medica, School of Pharmacy, North Sichuan Medical College, Nanchong, 637000 People’s Republic of China; 2 Department of Cardiology, Affiliated Hospital of North Sichuan Medical College, Nanchong, 637000 People’s Republic of China; 3 School of Clinical Medicine, North Sichuan Medical College, Nanchong, 637000 People’s Republic of China; 4 School of Preclinical Medicine, North Sichuan Medical College, and Nanchong Industry Technology Institute of BioMedicine, Nanchong, 637000 People’s Republic of China

**Keywords:** Danhong injection, Huangqi injection, Lipid, Dyslipidaemia, CHD

## Abstract

**Background:**

Dyslipidaemia is a major risk factor for coronary heart disease (CHD). Danhong and Huangqi injections, two traditional Chinese medicine prescriptions, have been widely studied regarding their lipid-lowering properties. However, the results were inconsistent and inconclusive. Thus, we conducted this meta-analysis of clinical controlled trials to clarify the lipid-lowering effects of Danhong and Huangqi injections.

**Methods:**

The databases including PubMed, Google Scholar, Web of Science, Cochrane Library, Wanfang Database, CNKI and VIP were searched. The following information was obtained from each study: first author, age, gender, ethnicity, health condition, treatment dose, treatment duration, sample size, mean and standard deviation or standard error of lipid variables before and after treatment. The changes in lipid levels from pre- to post-treatment were calculated and compared between the control groups and the treatment groups in this meta-analysis.

**Results:**

Forty-four studies (5021 subjects) and 7 studies (542 subjects) were respectively identified for Danhong and Huangqi injections. Compared with the control groups, Danhong injection yielded a significant reduction in triglycerides (TG) [standardized mean difference (SMD) = − 0.76, 95% confidence interval (CI) = (− 0.91, − 0.61), *P* <  0.001], total cholesterol (TC) [SMD = − 1.29, 95% CI = (− 1.56, − 1.03), *P* <  0.001] and low-density lipoprotein cholesterol (LDL-C) [SMD = − 0.76, 95% CI = (− 0.93, − 0.59), *P* <  0.001], and a significant elevation in high-density lipoprotein cholesterol (HDL-C) [SMD = 0.70, 95% CI = (0.41, 0.98), *P* <  0.001]. Regarding Huangqi injection, it yielded a significant reduction in TC [SMD = − 1.13, 95% CI = (− 2.09, − 0.16), *P* = 0.02] and marginally in TG [SMD = − 1.27, 95% CI = (− 2.53, 0.00), *P* = 0.05] comparing with the control groups.

**Conclusions:**

Danhong injection can effectively decrease the plasma levels of TG, TC and LDL-C, and increase HDL-C levels. Huangqi injection also has significant effects on TG and TC reduction, but not as powerful as Danhong injection.

**Electronic supplementary material:**

The online version of this article (10.1186/s12944-018-0760-2) contains supplementary material, which is available to authorized users.

## Background

Dyslipidaemia is a common lipid disorder characterized by increased levels of triglycerides (TG), total cholesterol (TC) and low-density lipoprotein cholesterol (LDL-C), and/or decreased level of high-density lipoprotein cholesterol (HDL-C) in circulation. Dyslipidaemia is one of the most important risk factors for coronary heart disease (CHD) and accounts for at least 50% of the population-attributable risk [1]. A review of randomized controlled trials (RCTs) concluded that a 40% reduction in LDL-C levels and a 30% increase in HDL-C levels could lower the CHD risk by 70% [2]. To date, several classes of lipid-lowering drugs have been introduced: inhibitors of 3-hydroxy-3-methylglutaryl coenzyme A reductase (e.g. statins), inhibitors of intestinal cholesterol absorption (e.g. ezetimibe), bile acid sequestrants (e.g. chlestyramine), anti-PCSK9 monoclonal antibodies (e.g. evolocumab), and so on, which have considerable benefits for improving serum lipid profiles but also have a number of drawbacks. For example, statins were reported to give rise to some serious adverse effects such as inflammatory and necrotizing myopathies [3–5]. Due to these concerns, there have been increasing attempts to use functional natural products as alternatives to the conventional lipid-modulating agents, which are often more acceptable to patients.

Danhong and Huangqi injections are two traditional Chinese medicines being widely used in clinical practice in China. Danhong injection is a herbal product from radix salviae miltiorrhizae and flos carthami. The main components of Danhong injection include tanshinone, salvia acid, salvianolic acid, safflower yellow pigment, safflower phenolic glycosides, catechol and so on. Clinical studies have shown that Danhong injection was effective in the treatment of CHD [6, 7], cerebral infarction [8] and hepatic veno-occlusive disease [9]. Huangqi injection (also known as astragalus injection) is made from radix astragali. Its main components are astragalosides, polysaccharides, flavones, amino acids and so on. Several researches concluded that Huangqi injection was effective in the treatment of aplastic anemia [10], diabetic nephropathy [11] and leucopenia [12]. The use of radix salviae miltiorrhizae, flos carthami and radix astragali was firstly described in the Chinese ancient medical books such as materia medica, Shen Nong Ben Cao Jing and Kai Bao Ben Cao. Currently, they are officially listed in the Chinese Pharmacopoeia.

A large number of studies have investigated the effects of Danhong and Huangqi injections on plasma lipid levels, but the results were inconsistent and inconclusive. In some of these studies, Danhong injection was reported to decrease plasma levels of TG [13–39], TC [16–43] and LDL-C [15, 17–33, 36–41, 44, 45], and increase HDL-C levels [17–32, 35, 40]; Huangqi injection was reported to decrease plasma levels of TG [46–51], TC [47–52] and LDL-C [48, 51], and increase HDL-C levels [51]. However, the results from other studies did not support these findings [53–56]. Therefore, a meta-analysis is required to clarify the lipid-lowering capacity of the two injections so that they can be better used in clinical practice. In the current study, we conducted a systematic review of randomized clinical trials to explore the effects of Danhong and Huangqi injections on plasma lipid levels. Our analysis results can provide a reference for future clinical practice.

## Results

### Characteristics of the included studies

The initial search of the databases yielded 5236 articles. Four thousand nine hundred and seventy-eight articles were excluded according to titles and abstracts. Then full-text articles were retrieved and assessed on the basis of the inclusion criteria. Two hundred and seven articles were ineligible for the following reasons: 187 articles did not provide lipid data; 12 articles provided incomplete lipid data; 8 articles just compared the lipid-lowering effects of Danhong or Huangqi injection with other drugs. In the end, 51 studies [13–63] were selected for this meta-analysis, and all of which were published in Chinese.

The characteristics of the 51 studies are summarized in Additional file 1: Table S1. Forty-four studies [13–45, 53–63] reported lipid data for Danhong injection, and 7 studies [46–52] reported lipid data for Huangqi injection. For Danhong injection, 38 studies [13–39, 42, 43, 54–62], 41 studies [13, 14, 16–43, 53–63], 38 studies [13–15, 17–34, 36–42, 44, 45, 53, 56–62] and 32 studies [13–15, 17–35, 37–40, 56–60, 62] presented the data for TG, TC, LDL-C and HDL-C, respectively. For Huangqi injection, 6 studies [46–51], 7 studies [46–52], 3 studies [46, 48, 51] and 3 studies [46, 48, 51] presented the data for TG, TC, LDL-C and HDL-C, respectively. Nine studies [15, 19, 20, 24, 27, 45, 54, 56, 60], 21 studies [17, 18, 21, 22, 25, 26, 28, 31, 32, 34, 35, 37, 38, 40, 41, 48, 57, 58, 61–63], 5 studies [13, 23, 33, 42, 59], 9 studies [14, 16, 29, 30, 36, 39, 43, 44, 55], 5 studies [47, 49–52] and 2 studies [46, 53] involved CHD, stroke, hyperlipidemia, diabetes, nephrotic syndrome and hypertension, respectively. Thirty studies [13, 16–25, 27–30, 34–36, 40, 42–45, 53–56, 58, 60, 63] and 14 studies [14, 15, 26, 31–33, 37–39, 41, 57, 59, 61, 62] respectively used high-dose treatment (> 20 mL) and low-dose treatment (≤ 20 mL) with Danhong injection. Four studies [46, 47, 49, 51] and 3 studies [48, 50, 52] respectively used high-dose treatment (≥ 40 mL) and low-dose treatment (< 40 mL) with Huangqi injection. Twenty studies [13, 15, 19–21, 23, 27, 30, 37–41, 43, 53, 56–58, 61, 62] and 23 studies [14, 16–18, 22, 24–26, 28, 29, 31–33, 35, 36, 42, 44, 45, 54, 55, 59, 60, 63] respectively used long-duration treatment (> 2 weeks) and short-duration treatment (≤ 2 weeks) with Danhong injection. Five studies [46–48, 51, 52] and 2 studies [49, 50] respectively used long-duration treatment (≥ 4 weeks) and short-duration treatment (< 4 weeks) with Huangqi injection. All the subjects included in the present meta-analysis were Chinese.

### Summary statistics

Five thousand and twenty-one subjects and 542 subjects were respectively enrolled in the analyses for Danhong and Huangqi injections. For Danhong injection, 49.7% of the subjects (2495 subjects) were controls, and 50.3% of them (2526 subjects) were treated with Danhong injection. For Huangqi injection, 49.3% of the subjects (267 subjects) were controls, and 50.7% of them (275 subjects) were treated with Huangqi injection. Four thousand two hundred and thirteen, 4781, 4412 and 3616 subjects were respectively included to compare the changes in TG, TC, LDL-C and HDL-C for Danhong injection (Aditional file 1: Table S1). Four hundred and forty, 542, 220 and 220 subjects were respectively included to compare the changes in TG, TC, LDL-C and HDL-C for Huangqi injection (Aditional file 1: Table S2).

### Associations of Danhong injection with plasma lipid levels

**Fig. 1:**
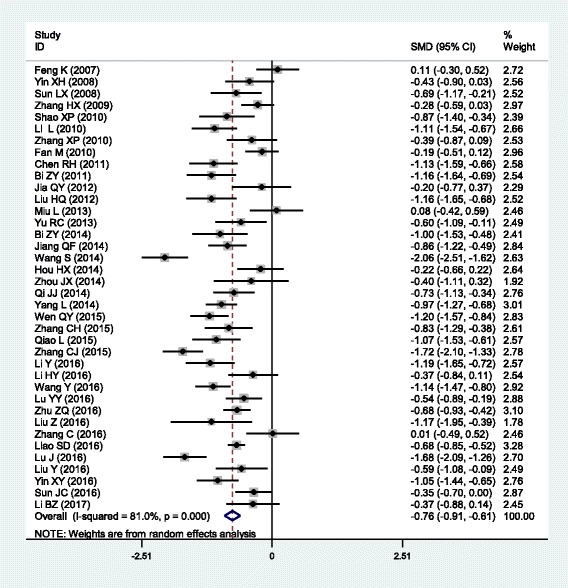
Forest plot of the meta-analysis between Danhong injection and the change in plasma TG levels

**Fig. 2:**
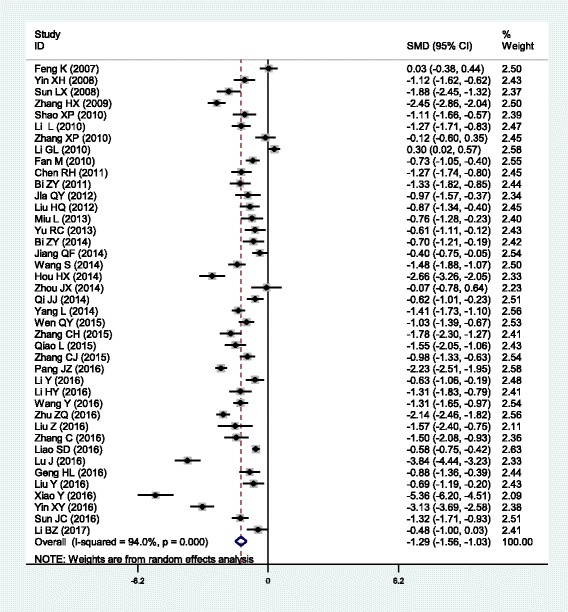
Forest plot of the meta-analysis between Danhong injection and the change in plasma TC levels

**Fig. 3:**
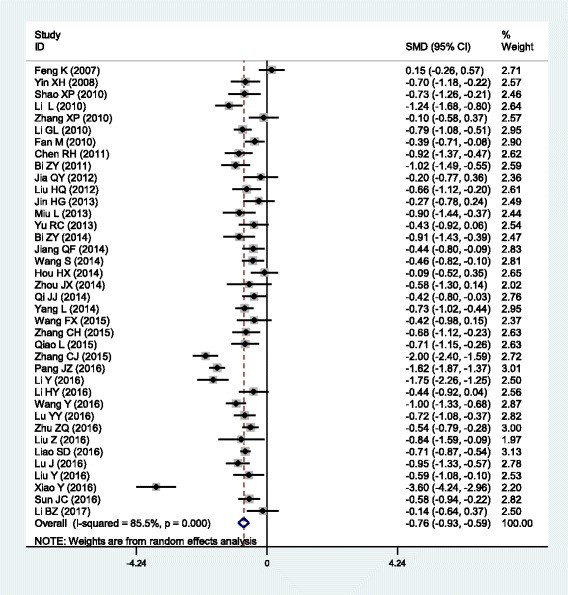
Forest plot of the meta-analysis between Danhong injection and the change in plasma LDL-C levels

**Fig. 4:**
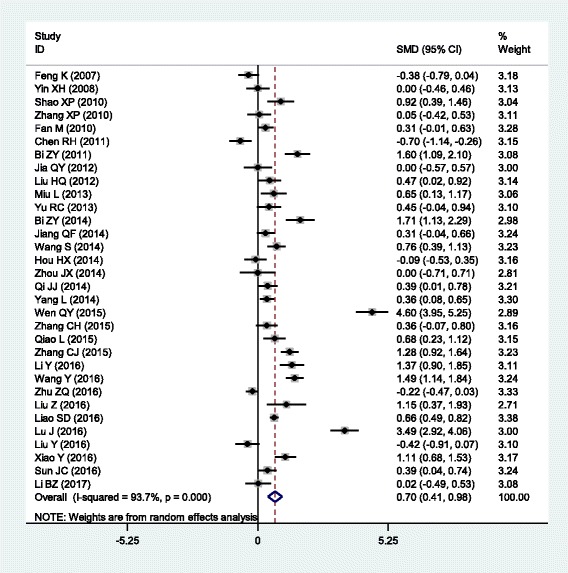
Forest plot of the meta-analysis between Danhong injection and the change in plasma HDL-C levels

The outcomes of the analysis on all studies showed that Danhong injection could effectively decrease the plasma levels of TG [SMD = − 0.77, 95% CI = (− 0.98, − 0.56), *P* <  0.001], TC [SMD = − 1.04, 95% CI = (− 1.30, − 0.78), *P* <  0.001] and LDL-C [SMD = − 0.69, 95% CI = (− 0.87, − 0.52), *P* <  0.001], and increase HDL-C levels [SMD = 0.47, 95% CI = (0.19, 0.75), *P* <  0.001] (Table 1 and Figs. 1, 2, 3 and 4).

**Table 1:** Meta-analysis of Danhong injection with the changes in plasma lipid levels

Groups or subgroups	Studies (Subjects)	*P*_Heterogeneity_	SMD (95% CI)	*P*_SMD_
Triglycerides				
All	38 (4213)	< 0.001	− 0.76 (− 0.91, − 0.61)	< 0.001
Coronary artery disease	7 (790)	< 0.001	− 0.52 (− 0.88, − 0.16)	< 0.01
Cerebrovascular disease	16 (2218)	< 0.001	− 0.94 (− 1.13, − 0.74)	< 0.001
Diabetes	9 (695)	0.001	− 0.58 (− 0.87, − 0.29)	< 0.001
Hyperlipidemia	5 (444)	< 0.001	− 0.87 (− 1.54, − 0.20)	< 0.05
Low-dose (≤ 20 mL)	13 (1257)	< 0.001	− 0.90 (− 1.15, − 0.66)	< 0.001
High-dose (> 20 mL)	25 (2956)	< 0.001	− 0.69 (− 0.88, − 0.50)	< 0.001
Short-duration (≤ 2 weeks)	20 (2472)	< 0.001	− 0.76 (− 0.99, − 0.53)	< 0.001
Long-duration (> 2 weeks)	17 (1661)	< 0.001	− 0.80 (− 0.99, − 0.61)	< 0.001
Total cholesterol				
All	41 (4781)	< 0.001	− 1.29 (− 1.56, − 1.03)	< 0.001
Coronary artery disease	7 (982)	< 0.001	− 1.12 (− 1.85, − 0.40)	< 0.01
Cerebrovascular disease	18 (2390)	< 0.001	− 1.53 (− 1.94, − 1.13)	< 0.001
Diabetes	9 (695)	< 0.001	− 1.28 (− 1.87, − 0.70)	< 0.001
Hyperlipidemia	6 (648)	0.08	−1.16 (− 1.46, − 0.86)	< 0.001
Low-dose (≤ 20 mL)	13 (1449)	< 0.001	− 1.13 (− 1.49, − 0.78)	< 0.001
High-dose (> 20 mL)	28 (3332)	< 0.001	− 1.38 (− 1.73, − 1.02)	< 0.001
Short-duration (≤ 2 weeks)	21 (2544)	< 0.001	− 1.12 (− 1.38, − 0.86)	< 0.001
Long-duration (> 2 weeks)	19 (2157)	< 0.001	− 1.42 (− 1.92, − 0.92)	< 0.001
LDL-C				
All	38 (4412)	< 0.001	− 0.76 (− 0.93, − 0.59)	< 0.001
Coronary artery disease	8 (1012)	< 0.001	− 0.72 (− 1.15, − 0.30)	0.001
Cerebrovascular disease	16 (2183)	< 0.001	− 0.94 (− 1.24, − 0.64)	< 0.001
Diabetes	7 (503)	0.02	− 0.56 (− 0.86, − 0.26)	< 0.001
Hyperlipidemia	5 (444)	0.34	−0.54 (− 0.74, − 0.34)	< 0.001
Low-dose (≤ 20 mL)	14 (1579)	< 0.001	− 0.79 (− 1.05, − 0.53)	< 0.001
High-dose (> 20 mL)	24 (2833)	< 0.001	− 0.74 (− 0.96, − 0.52)	< 0.001
Short-duration (≤ 2 weeks)	18 (2115)	< 0.001	− 0.71 (− 0.92, − 0.50)	< 0.001
Long-duration (> 2 weeks)	19 (2217)	< 0.001	−0.85 (− 1.11, − 0.58)	< 0.001
HDL-C				
All	32 (3616)	< 0.001	0.70 (0.41, 0.98)	< 0.001
Coronary artery disease	5 (500)	< 0.001	0.75 (−0.00, 1.50)	0.05
Cerebrovascular disease	17 (2318)	< 0.001	0.98 (0.55, 1.41)	< 0.001
Diabetes	4 (288)	0.10	0.32 (−0.07, 0.70)	0.10
Hyperlipidemia	5 (444)	< 0.001	0.19 (−0.32, 0.69)	0.47
Low-dose (≤ 20 mL)	11 (1032)	< 0.01	0.53 (0.33, 0.73)	< 0.001
High-dose (> 20 mL)	21 (2584)	< 0.001	0.79 (0.38, 1.21)	< 0.001
Short-duration (≤ 2 weeks)	16 (2070)	< 0.001	0.92 (0.55, 1.29)	< 0.001
Long-duration (> 2 weeks)	15 (1466)	< 0.001	0.51 (0.07, 0.95)	0.02

*SMD* standardized mean difference, *95% CI* 95% confidence interval, *TG* triglycerides, *TC* total cholesterol, *LDL-C* low-density lipoprotein cholesterol, *HDL-C* high-density lipoprotein cholesterol

In the subgroup analyses stratified by health status, Danhong injection had significant effects on all of the four lipid parameters in CHD patients [TG: SMD = − 1.04, 95% CI = (− 1.30, − 0.78), *P* <  0.001; TC: SMD = − 1.04, 95% CI = (− 1.30, − 0.78), *P* <  0.001; LDL-C: SMD = − 1.04, 95% CI = (− 1.30, − 0.78), *P* <  0.001 and HDL-C: SMD = − 1.04, 95% CI = (− 1.30, − 0.78), *P* <  0.001] and in stroke patients [TG: SMD = − 0.94, 95% CI = (− 1.13, − 0.74), *P* <  0.001; TC: SMD = − 1.53, 95% CI = (− 1.94, − 1.13), *P* <  0.001; LDL-C: SMD = − 0.94, 95% CI = (− 1.24, − 0.64), *P* <  0.001; HDL-C: SMD = 0.98, 95% CI = (0.55, 1.41), *P* <  0.001]. Danhong injection could significantly decrease TG, TC and LDL-C levels, but not HDL-C levels in diabetic patients [TG: SMD = − 0.58, 95% CI = (− 0.87, − 0.29), *P* <  0.001; TC: SMD = − 1.28, 95% CI = (− 1.87, − 0.70), *P* <  0.001; LDL-C: SMD = − 0.56, 95% CI = (− 0.86, − 0.26), *P* <  0.001] and in hyperlipidemic patients [TG: SMD = − 0.87, 95% CI = (− 1.54, − 0.20), *P* <  0.001; TC: SMD = − 1.16, 95% CI = (− 1.46, − 0.86), *P* <  0.001; LDL-C: SMD = − 0.54, 95% CI = (− 0.74, − 0.34), *P* <  0.001].

In the subgroup analyses stratified by the dose of the treatment with Danhong injection, both low- and high-dose of treatment could significantly decrease the levels of TG [low-dose: SMD = − 0.90, 95% CI = (− 1.15, − 0.66), *P* <  0.001; high-dose: SMD = − 0.69, 95% CI = (− 0.88, − 0.50), *P* <  0.001], TC [low-dose: SMD = − 1.13, 95% CI = (− 1.49, − 0.78), *P* <  0.001; high-dose: SMD = − 1.38, 95% CI = (− 1.73, − 1.02), *P* <  0.001] and LDL-C [low-dose: SMD = − 0.79, 95% CI = (− 1.05, − 0.53), *P* <  0.001; high-dose: SMD = − 0.74, 95% CI = (− 0.96, − 0.52), *P* <  0.001], and increase HDL-C levels [low-dose: SMD = 0.53, 95% CI = (0.33, 0.73), *P* <  0.001; high-dose: SMD = 0.79, 95% CI = (0.38, 1.21), *P* <  0.001]. In the subgroup analyses stratified by the duration of the treatment with Danhong injection, both short- and long-duration of treatment could significantly decrease the levels of TG [short-duration: SMD = − 0.76, 95% CI = (− 0.99, − 0.53), *P* <  0.001; long-duration: SMD = − 0.80, 95% CI = (− 0.99, − 0.61), *P* <  0.001], TC [short-duration: SMD = − 1.12, 95% CI = (− 1.38, − 0.86), *P* <  0.001; long-duration: SMD = − 1.42, 95% CI = (− 1.92, − 0.92), *P* <  0.001] and LDL-C [short-duration: SMD = − 0.71, 95% CI = (− 0.92, − 0.50), *P* <  0.001; long-duration: SMD = − 0.85, 95% CI = (− 1.11, − 0.58), *P* <  0.001], and increase HDL-C levels [short-duration: SMD = 0.92, 95% CI = (0.55, 1.29), *P* <  0.001; long-duration: SMD = 0.51, 95% CI = (0.07, 0.95), *P* = 0.02].

### Associations of Huangqi injection with plasma lipid levels

**Fig. 5:**
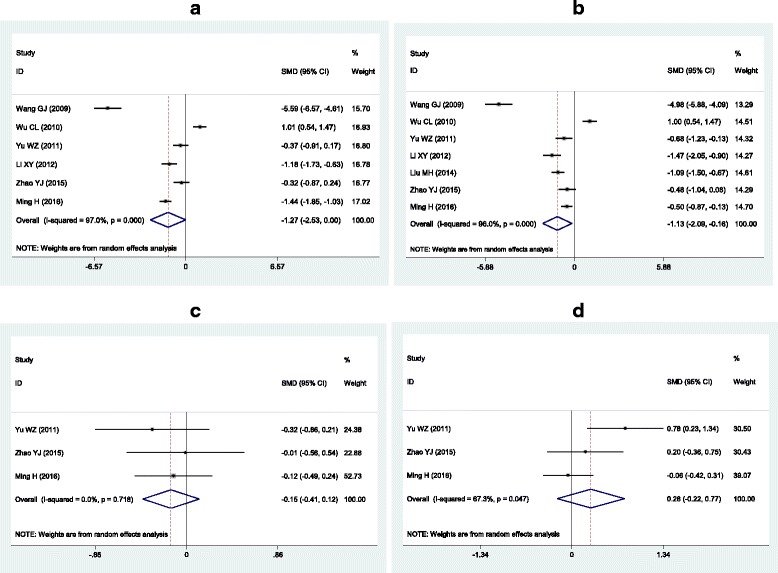
Forest plots of the meta-analysis between Huangqi injection and the changes in plasma lipid levels. **a** Forest plot of the meta-analysis between Huangqi injection and the change in plasma TG levels; **b** Forest plot of the meta-analysis between Huangqi injection and the change in plasma TC levels; **c** Forest plot of the meta-analysis between Huangqi injection and the change in plasma LDL-C levels; **d** Forest plot of the meta-analysis between Huangqi injection and the change in plasma HDL-C levels

The outcomes of the analysis on all studies showed that Huangqi injection could significantly decrease TC levels [SMD = − 1.13, 95% CI = (− 2.09, − 0.16), *P* = 0.02], and marginally significantly decrease TG levels [SMD = − 1.27, 95% CI = (− 2.53, 0.00), *P* = 0.05]. Huangqi injection did not have significant effects on the changes in LDL-C and HDL-C levels (Table 2 and Fig. 5).

**Table 2:** Meta-analysis of Huangqi injection with the changes in plasma lipid levels

Groups or subgroups	Studies (Subjects)	*P*_Heterogeneity_	SMD (95% CI)	*P*_SMD_
TG				
All	6 (440)	< 0.001	−1.27 (− 2.53, 0.00)	0.05
Nephrotic syndrome	4 (274)	< 0.001	−1.49 (− 3.56, 0.58)	0.16
Low-dose (< 40 mL)	2 (196)	< 0.001	−3.49 (− 7.55, 0.57)	0.09
High-dose (≥ 40 mL)	4 (244)	< 0.001	−0.21 (−1.14, 0.73)	0.67
Short-duration (< 4 weeks)	2 (160)	< 0.001	−2.28 (−8.74, 4.19)	0.49
Long-duration (≥ 4 weeks)	4 (280)	< 0.01	−0.84 (−1.43, − 0.26)	< 0.01
TC				
All	7 (542)	< 0.001	−1.13 (−2.09, −0.16)	0.02
Nephrotic syndrome	5 (376)	< 0.001	−1.41 (−2.88, 0.07)	0.06
Low-dose (< 40 mL)	3 (298)	< 0.001	−2.13 (−3.98, −0.29)	0.02
High-dose (≥ 40 mL)	4 (244)	< 0.001	−0.40 (−1.47, 0.67)	0.47
Short-duration (< 4 weeks)	2 (160)	< 0.001	−1.98 (−7.85, 3.89)	0.51
Long-duration (≥ 4 weeks)	5 (382)	0.03	−0.83 (−1.19, − 0.47)	< 0.001
LDL-C				
All	3 (220)	0.72	−0.15 (− 0.41, 0.12)	0.28
HDL-C				
All	3 (220)	< 0.05	0.28 (−0.22, 0.77)	0.27

*SMD* standardized mean difference, *95% CI* 95% confidence interval, *TG* triglycerides, *TC* total cholesterol, *LDL-C* low-density lipoprotein cholesterol, *HDL-C* high-density lipoprotein cholesterol

### Heterogeneity analysis

In the analysis for Danhong injection, there was significant heterogeneity in the total analyses for TG, TC, LDL-C and HDL-C (Table 1). Thirteen studies [13, 22, 24, 29, 33–35, 54–56, 58–60], 20 studies [14, 16, 17, 21, 26–29, 34, 39–43, 53, 54, 56–58, 62], 9 studies [14, 22, 26, 28, 29, 34, 40, 41, 56] and 15 studies [17–26, 34, 35, 40, 56, 57] were respectively identified as the main contributors to the heterogeneity for TG, TC, LDL-C and HDL-C by using Galbraith plots (Additional file 2: Figures S1-S4). The heterogeneity was effectively removed or decreased after exclusion of these outlier studies, but the SMD values and 95% CIs did not change substantially [TG: SMD = − 0.80, 95% CI = (− 0.90, − 0.70), *P*_SMD_ <  0.001, *P*_Heterogeneity_ = 0.06; TC: SMD = − 1.17, 95% CI = (− 1.28, − 1.06), *P*_SMD_ <  0.001, *P*_Heterogeneity_ = 0.17; LDL-C: SMD = − 0.68, 95% CI = (− 0.76, − 0.61), *P*_SMD_ <  0.001, *P*_Heterogeneity_ = 0.26; HDL-C: SMD = 0.42, 95% CI = (0.29, 0.54), *P*_SMD_ <  0.001, *P*_Heterogeneity_ = 0.17].

In the analysis for Huangqi injection, there was significant heterogeneity in the total analyses for TG, TC and HDL-C (Table 2). Three studies [48–50], 3 studies [47, 49, 50] and 1 study [51] were respectively identified as the main contributors to the heterogeneity for TG, TC and HDL-C by using Galbraith plots (Additional file 2: Figures S5-S7). The heterogeneity was effectively removed or decreased after exclusion of these outlier studies, but the SMD values and 95% CIs did not change substantially [TG: SMD = − 0.62, 95% CI = (− 1.17, − 0.08), *P*_SMD_ = 0.03, *P*_Heterogeneity_ = 0.051; TC: SMD = − 0.70, 95% CI = (− 1.00, − 0.40), *P*_SMD_ <  0.001, *P*_Heterogeneity_ = 0.17; HDL-C: SMD = − 0.02, 95% CI = (− 0.29, 0.33), *P*_SMD_ = 0.46, *P*_Heterogeneity_ = 0.90].

### Publication bias test

Begg’s test and funnel plot were used to detect the potential publication bias, and no evidence of significant publication bias was detected in the analyses for Danhong injection (TG: *Z* = 0.65, *P* = 0.51; TC: *Z* = 1.74, *P* = 0.08; LDL-C: *Z* = 0.03, *P* = 0.98; HDL-C: *Z* = 1.48, *P* = 0.14) (Additional file 2: Figures S8-S11), and for Huangqi injection (TG: *Z* = 0.38, *P* = 0.71; TC: *Z* = 1.50, *P* = 0.13; LDL-C: *Z* = 0.00, *P* = 1.00; HDL-C: *Z* = 0.00, *P* = 1.00) (Additional file 2: Figures S12-S15).

## Discussion

A substantial number of clinical trials have investigated the lipid-lowering effects of Danhong and Huangqi injections. Associations of the two injections with decreased levels of TG, TC and LDL-C, and/or increased level of HDL-C have been reported in some, but not all studies. The lack of consistency across these studies reflects some limitations such as small sample size and differences in dose and duration of treatment. In the present meta-analysis, the lipid-lowering effects of Danhong and Huangqi injections were investigated to clarify these discrepancies. To our knowledge, the present study is the first meta-analysis to explore the lipid-lowering effects of Danhong and Huangqi injections based on RCTs.

The results of this meta-analysis suggested that Danhong injection could effectively decrease plasma TG, TC and LDL-C levels, and increase HDL-C level in the total population. In subgroup analyses, we found that Danhong injection could decrease TG, TC, LDL-C, and increase HDL-C levels in CHD and stroke patients. In diabetic and hyperlipidemic patients, Danhong injection could significantly decrease TG, TC and LDL-C levels, but had no significant effect on HDL-C level. The lipid-lowering effects of Danhong injection were very robust, which did not vary greatly when the analysis was stratified by dose and duration of treatment. The present meta-analysis also demonstrated that Huangqi injection could decrease plasma levels of TG and TC, but the effects of Huangqi injection on plasma lipid levels need to be further explored with large sample size.

The mechanisms by which Huangqi injection lowers plasma lipid levels have not been clarified yet. However, several studies [64, 65] have investigated the potential mechanisms by which Danhong injection reduced plasma lipid levels. Chen J et al. [64] demonstrated that the lipid-lowering effects of Danhong injection were mediated by up-regulation of the lipolytic genes including carnitine palmitoyl transferase 1 (*CPT1*) and peroxisome proliferator-activated receptor alpha (*PPARA*), and down-regulation of the lipogenic genes including fatty acid synthase (*FAS*) and hydroxymethylglutaryl-CoA reductase (*HMGCR*). The upregulation of adenosine triphosphate-binding cassette transporter A1 (*ABCA1*), a key factor in reverse cholesterol transport pathway, by Danhong injection was also reported [65].

The duration of the treatment with Danhong and Huangqi injections was varied from 1 week to 6 months in the present meta-analysis. By searching the databases such as PubMed, Google Scholar, Web of Science, Cochrane Library, Wanfang Database, CNKI and VIP, we did not find any studies which had the treatment duration longer than 6 months. By analyzing the original data from the studies included in the present meta-analysis, we found that the treatment duration of Danhong injection had profound effects on blood lipid levels. For example, Danhong injection could decrease TG, TC and LDL-C by 12–17% after 2 weeks of treatment, 20–25% after 1 to 2 months of treatment and remained at these levels thereafter. It could increase HDL-C by 14–16% after 2 weeks of treatment, 21–24% after 1 to 2 months of treatment, and 37–40% after 5 to 6 months of treatment. Regarding Huangqi injection, it could decrease TG, TC and LDL-C by 8–13% after 2 weeks to 1 month of treatment, and 8–18% after 6 months of treatment. Huangqi injection had no effect on HDL-C levels.

None of the included studies in the present meta-analysis has mentioned the side effects of Danhong injection. However, one study [49] pointed out that Huangqi injection had a side effect of xerostomia in a few patients. In addition, several studies [66–69] which specifically focused on the side effects of Danhong and Huangqi injections demonstrated that there might be some side effects of Danhong injection such as skin pruritus, flushing, rash, injection site swelling, nausea and vomiting, and of Huangqi injection such as vomiting, rash, fever, dyspnea and bosom frowsty. No toxicology has been reported for Danhong or Huangqi injection.

U.S. Food and Drug Administration (USFDA) promulgated Good Laboratory Practices (GLP) in 1979, and the guidelines of which gradually became the international standards to ensure uniformity, consistency, reliability, reproducibility, quality, and integrity of chemical (including pharmaceuticals) non-clinical safety tests. Based on the GLP guidelines of USFDA and combined with the actual situations in China, China Food and Drug Administration (CFDA) promulgated Chinese GLP in 2003, and last updated in September, 2017 to ensure drug safety and to avoid the occurrence of drug damage to the greatest extent. Chinese GLP has a set of strict guidelines for drugs such as new drug approval process, adverse drug reaction monitoring and drug instruction supervision. The certification steps of new drugs in China include: application, acceptance, data review, on-site inspection, audit, announcement and so on. In the process of clinical use, the government and the relevant functional departments will also follow up on the safety, efficacy, rational drug use and combined drug use. Danhong and Huangqi injections had to fulfill all the demands and guidelines mentioned above before they could be put into clinical use. The Danhong injection (national medicine permission number: Z20026866; national quality standard: ≥ 5 mg total flavonoids per mL) in the present meta-analysis was mainly made by Ji’nan Buchang Pharmaceutical Co., Ltd. P. R. China, Heze Buchang Pharmaceutical Co., Ltd. P. R. China and Xianyang Buchang Pharmaceutical Co., Ltd. P. R. China. The Huangqi injection (national medicine permission number: Z20003189; national quality standard: ≥ 2 mg astragaloside IV per mL) in the present meta-analysis was mainly made by Chengdu Di’ao Jiuhong Pharmaceutical Co., Ltd. P. R. China, Jiangsu Jiuxu Pharmaceutical Co., Ltd. P. R. China and Shijiazhuang Shenwei Pharmaceutical Co., Ltd. P. R. China.

With regard to lipid-lowering effects, there was no study that compared Huangqi injection with statins or other drugs. However, two studies [20, 70] investigated the lipid-lowering effects of Danhong injection as compared with statins. In a hospital-based study, Bi ZY [20] demonstrated that the lipid-lowering abilities of Danhong injection (40 mL/d) were very similar to those of atorvastatin (10 mg/d), in which TC, LDL-C and TG were significantly reduced, and HDL-C was significantly increased to the same degree with both drugs after 6 months of treatment. Fan HJ et al. [70] compared the lipid-lowering abilities of Danhong injection (at doses of 1.0 mL/kg, 2.0 mL/kg, 4.0 mL/kg) with simvastatin (2.0 mg/kg) in rats and found that at the dose of 4.0 mL/kg, Danhong injection had comparable lipid-lowering effects as simvastatin.

Significant heterogeneity was detected in the analysis for Danhong injection (TG, TC, LDL-C and HDL-C) and Huangqi injection (TG and TC). Subgroup analysis stratified by the health status of subjects, dose and duration of treatment was performed to explore the potential sources of the observed heterogeneity, and the results showed that the main sources of heterogeneity were from the health status of subjects, dose and duration of treatment. Galbraith plot was employed to figure out the studies which produced heterogeneity. Outlier studies were identified by using the plots, and heterogeneity was effectively removed or decreased after excluding the outlier studies. No significant changes in SMD values and 95% CIs were found after excluding the outlier studies. The results from this meta-analysis were based on random effects model. Comparing with fixed effects model, the random effects model is a more conservative method and less likely to produce false-positive results. Funnel plots and Begg’s tests showed no publication bias for all of the lipid parameters.

Several limitations should be acknowledged in this meta-analysis. Firstly, all the included studies used an A versus A + B design in which patients were randomized to receive a control treatment (control group) or a control treatment plus an experimental treatment (treatment group). This kind of design is likely to generate false positive results [71]. However, it was not possible to find well designed trials to evaluate the lipid-lowering effects of Danhong or Huangqi injection. Secondly, a relatively small number of subjects were included in the analysis for Huangqi injection, which may reduce the statistic power and even cause type I error (false-positive results). Further studies with large sample size are required to investigate the lipid-lowering effects of Huangqi injection. Thirdly, this meta-analysis only included the studies published in Chinese, and all of the subjects included were Chinese as there were no studies being conducted outside of China so far. Due to this limitation, the results of this study may only apply to Chinese populations, but cannot be extended to populations elsewhere.

In summary, the present meta-analysis demonstrated that Danhong injection can reduce plasma levels of TG, TC and LDL-C and increase HDL-C level. Huangqi injection also has significant effects on TG and TC, but not as powerful as Danhong injection.

## Methods

### Identification and eligibility of relevant studies

All articles published before April 2017 on the effects of Danhong and Huangqi injections on plasma lipid levels were identified. The languages of the articles were limited to English and Chinese. A comprehensive search of the literature was carried out by using the databases including PubMed, Google Scholar, Web of Science, Cochrane Library, Wanfang, CNKI and VIP. The keywords used for this search were “Danhong or Huangqi or Danhong injection or Huangqi injection or astragalus injection” concatenated with “triglyceride or total cholesterol or high-density lipoprotein cholesterol or low-density lipoprotein cholesterol or TG or TC or HDL-C or LDL-C or hyperlipidemia or dyslipidaemia or hypercholesterolemia or hypertriglyceridemia or coronary heart disease or coronary artery disease or stroke or diabetes”. The variables of this meta-analysis were limited to TG, TC, LDL-C and HDL-C. The studies that fulfilled the following criteria were included: (1) studies in which an A versus A + B design was used, i.e. a control treatment (control group) versus a control treatment plus an experimental treatment (treatment group); (2) studies being designed as randomized and double-blind clinical trials; (3) studies reporting the effects of Danhong or Huangqi injection on at least one of the four lipid variables (TG, TC, LDL-C and HDL-C); (4) studies in which mean values and standard deviations (SD) or standard errors (SE) were available; (5) studies in which fasting lipid levels were given; (6) lipid data were available before and after the treatment of Danhong or Huangqi injection; (7) lipid data were available for both control group and treatment group. All references cited in the included articles were reviewed to check the published work which was not indexed by PubMed, Google Scholar, Web of Science, Cochrane Library, Wanfang, CNKI and VIP databases. Reports with incomplete data, studies based on pedigree data, case reports, review articles, abstracts and animal studies were excluded from the meta-analysis.

### Data extraction

Data were extracted by using a structured data collection form. The irrelevant studies or the studies that did not meet the inclusion criteria were excluded after being reviewed independently by two reviewers. The data were double-checked and compared after extraction. Data uncertainty was discussed and solved by the whole group. For the overlapping articles, only those publications that presented the most detailed information were included. In the present meta-analysis, the data extracted from each of the included studies were as follows: first author, age, gender, ethnicity, health condition, treatment dose, treatment duration, sample size, lipid data before and after the treatment of Danhong or Huangqi injection.

### Statistical analysis

The unit mmol/L was used for all lipid variables in the meta-analysis. The standardized mean difference (SMD) for net change and 95% confidence interval (95% CI) were used to determine the effects of Danhong or Huangqi injection on plasma levels of TG, TC, LDL-C and HDL-C. The following formula was used to calculate the changes in mean values from pre- to post-treatment: mean change = mean_post-treatment_ - mean_pre-treatment_, and the following formula was used to calculate the changes in SD values: SD = square root [(SD_pre-treatment_)^2^ + (SD_post-treatment_)^2^ – 2R × SD_pre-treatment_ × SD_post-treatment_], assuming a correlation coefficient (R) = 0.5 [72]. The STATA software package (Version 10, Stata Corporation, College Station, TX) was used for all statistical analyses. All data were presented as mean ± SD in this meta-analysis. Subgroup analyses were conducted according to dose of treatment, duration of treatment and health conditions. Health condition subgroup was defined as CHD, diabetes, stroke and hyperlipidemia.

The random effects model was used in the meta-analysis in that (1) both between-study and within-study heterogeneity is considered in this model; (2) the random effects model provides a more conservative evaluation of the significance of the associations than the fixed effects model [73]. Heterogeneity among studies was tested by Cochran’s χ^2^-based Q-statistic at a significance level of *P* <  0.05. Galbraith plot was used to detect the potential sources of heterogeneity, and the SMD values were recalculated after excluding the outlier studies. Publication bias was assessed by Begg’s rank correlation tests and funnel plots, and a significance level of 0.05 was used to indicate the presence of potential publication bias [74].

## Additional files

Additional file 1: **Table S1.** Characteristics of the studies included in the meta-analysis for Danhong injection; **Table S2.** Characteristics of the studies included in the meta-analysis for Huangqi injection; **Table S3.** The lipid level changes from pre- to post-treatment with Danhong injection in control or treatment groups; **Table S4.** The lipid level changes from pre- to post-treatment with Huangqi injection in control or treatment groups. (DOCX 65 kb)

Additional file 2: **Figures S1-S4.** Galbraith plots of the association analysis between Danhong injection and the changes in plasma levels of TG, TC, LDL-C and HDL-C, respectively; **Figures S5-S7.** Galbraith plots of the association analysis between Huangqi injection and the changes in plasma levels of TG, TC and HDL-C, respectively; **Figures S8-S11.** Begg’s funnel plots of the association analysis between Danhong injection and the changes in plasma levels of TG, TC, LDL-C and HDL-C, respectively; **Figures S12-S15.** Begg’s funnel plots of the association analysis between Huangqi injection and the changes in plasma levels of TG, TC, LDL-C and HDL-C, respectively. (DOCX 54 kb)

## Abbreviations

95% CI: 95% confidence interval
CHD: Coronary heart disease
HDL-C: High-density lipoprotein cholesterol
LDL-C: Low-density lipoprotein cholesterol
SMD: Standardized mean difference
TC: Total cholesterol
TG: Triglycerides
