# Supplementary material for: The lipid-lowering effects of Danhong and Huangqi injections: a meta-analysis of clinical controlled trials
Source: Lipids Health Dis. 2018 May 10;17:106. doi: 10.1186/s12944-018-0760-2 (PMC5944065; doi:10.1186/s12944-018-0760-2)
Supplement: Supplementary file 1 — Table S1. Characteristics of the studies included in the meta-analysis for Danhong injection; Table S2. Characteristics of the studies included in the meta-analysis for Huangqi injection; Table S3. The lipid level changes from pre- to post-treatment with Danhong injection in control or treatment groups; Table S4. The lipid level changes from pre- to post-treatment with Huangqi injection in control or treatment groups. (DOCX 65 kb) [file 12944_2018_760_MOESM1_ESM.docx]

**Supplemental Tables**

**Table S1.** Characteristics of the studies included in the meta-analysis for Danhong injection.

**Table S2.** Characteristics of the studies included in the meta-analysis for Huangqi injection.

**Table S3.** The lipid level changes from pre- to post-treatment with Danhong injection in control or treatment groups.

**Table 4.** The lipid level changes from pre- to post-treatment with Huangqi injection in control or treatment groups.

**Table S1. Characteristics of the studies included in the meta-analysis for Danhong injection.**

| First author,  reference | Year | Ethnicity | Gender | Dose (mL) | Age (year)  control/treatment | Study duration | Numbers  Control/treatment | Study population | Outcomes |
| --- | --- | --- | --- | --- | --- | --- | --- | --- | --- |
| Jia QY [13] | 2012 | Chinese | M/F | 40 | >50.0 | 4 weeks | 24/24 | Hyperlipidemia | TG/TC/LDL-C/HDL-C |
| Zhang XP [14] | 2010 | Chinese | M/F | 20 | 67.7±8.5 | 14 days | 34/34 | Type 2 diabetes mellitus | TG/TC/LDL-C/HDL-C |
| Lu YY [15] | 2016 | Chinese | M/F | 20 | 64.4±4.0/62.4±3.7 | 15 days | 65/65 | Coronary artery disease | TG/LDL-C |
| Yin XY [16] | 2016 | Chinese | M/F | 30 | 57.7±4.9/59.5±5.6 | 2 weeks | 56/56 | Diabetic nephropathy | TG/TC |
| Liao SD [17] | 2016 | Chinese | M/F | 40 | 73.0-88.0/65.0-90.0 | 7-10 days | 300/300 | Cerebrovascular disease | TG/TC/LDL-C/HDL-C |
| Bi ZY [18] | 2011 | Chinese | M/F | 30 | 65.5±3.0/65.2±3.5 | 14 days | 40/40 | Cerebrovascular disease | TG/TC/LDL-C/HDL-C |
| Liu Y [19] | 2016 | Chinese | M/F | 40 | 57.4±13.1/55.8±12.7 | 2 weeks | 33/33 | chronic cor pulmonale | TG/TC/LDL-C/HDL-C |
| Bi ZY [20] | 2014 | Chinese | M/F | 40 | 62.1±3.2/61.7±3.8 | 6 months | 30/33 | Coronary artery disease | TG/TC/LDL-C/HDL-C |
| Zhu ZQ [21] | 2016 | Chinese | M/F | 30 | 55.1±10.9/54.6±11.2 | 8 weeks | 121/121 | Cerebrovascular disease | TG/TC/LDL-C/HDL-C |
| Zhang CJ [22] | 2015 | Chinese | M/F | 40 | 58.0±9.5/58.5±10.2 | 2 weeks | 72/70 | Cerebrovascular disease | TG/TC/LDL-C/HDL-C |
| Chen RH [23] | 2011 | Chinese | M/F | 30 | 42.0-74.0/40.0-75.0 | 15 days | 42/42 | Hyperlipidemia | TG/TC/LDL-C/HDL-C |
| Wang Y [24] | 2016 | Chinese | M/F | 30 | 65.9±3.7/65.3±3.6 | 14 days | 80/80 | Coronary artery disease | TG/TC/LDL-C/HDL-C |
| Yin XH [25] | 2008 | Chinese | M/F | 30 | 64.0-79.0/65.0-78.0 | 14 days | 36/36 | Cerebrovascular disease | TG/TC/LDL-C/HDL-C |
| Li Y [26] | 2016 | Chinese | M/F | 20 | 60.6±10.8/61.2±9.8 | 14 days | 42/42 | Cerebrovascular disease | TG/TC/LDL-C/HDL-C |
| Jiang QF [27] | 2014 | Chinese | M/F | 30 | 65.4±5.1 | 8 weeks | 63/63 | Coronary artery disease | TG/TC/LDL-C/HDL-C |
| Li BZ [28] | 2017 | Chinese | M/F | 40 | 62.0±4.1/61.5±4.0 | 14 days | 30/30 | Cerebrovascular disease | TG/TC/LDL-C/HDL-C |
| Fan M [29] | 2010 | Chinese | M/F | 30-50 | 57.0-85.0/56.0-91.0 | 2 weeks | 67/92 | Diabetic nephropathy | TG/TC/LDL-C/HDL-C |
| Liu Z [30] | 2016 | Chinese | M/F | 20-40 | 60.2±5.1/59.9±5.2 | 3 weeks | 15/15 | Diabetic nephropathy | TG/TC/LDL-C/HDL-C |
| Shao XP [31] | 2010 | Chinese | M/F | 20 | 40.0-80.0/41.0-82.0 | 14 days | 30/30 | Cerebrovascular disease | TG/TC/LDL-C/HDL-C |
| Liu HQ [32] | 2012 | Chinese | M/F | 20 | 59.2±7.15/57.5±8.26 | 2 weeks | 43/35 | Cerebrovascular disease | TG/TC/LDL-C/HDL-C |
| Wang S [33] | 2014 | Chinese | M/F | 20 | 56.4±10.2 | 14 days | 60/60 | Hyperlipidemia | TG/TC/LDL-C/HDL-C |
| Hou HX [34] | 2014 | Chinese | M/F | 20-40 | 64.2±3.9/67.9±5.2 | - | 40/40 | Cerebrovascular disease | TG/TC/LDL-C/HDL-C |
| Wen QY[35] | 2015 | Chinese | M/F | 40 | 59.7±17.0/61.4±18.5 | 14 days | 67/68 | Cerebrovascular disease | TG/TC/LDL-C |
| Li HY [36] | 2016 | Chinese | M/F | 40 | 59.4±2.1 | 2 weeks | 40/30 | Type 2 diabetes mulletus | TG/TC/LDL-C |
| Qiao L [37] | 2015 | Chinese | M/F | 20 | 62.5±5.3/61.2±5.9 | 8 weeks | 41/41 | Cerebrovascular disease | TG/TC/LDL-C/HDL-C |
| Yang L [38] | 2014 | Chinese | M/F | 20 | 59.4±11.6 | 2 months | 98/98 | Cerebrovascular disease | TG/TC/LDL-C/HDL-C |
| Zhou JX [39] | 2014 | Chinese | M/F | 20 | 54.6±8.6/55.5±9.2 | 4 weeks | 14/17 | Diabetic nephropathy | TG/TC/LDL-C/HDL-C |
| Xiao Y [40] | 2016 | Chinese | M/F | 20-30 | 60.2±4.7/57.3±2.9 | 1 month | 50/50 | Cerebrovascular disease | TC/LDL-C/HDL-C |
| Pang JZ [41] | 2016 | Chinese | M/F | 20 | 63.3±3.2 | 28 days | 159/163 | Coronary artery disease | TC/LDL-C |
| Yu RC [42] | 2013 | Chinese | M/F | 40 | 62.4±14.0/58.1±17.7 | 2 weeks | 32/34 | Hyperlipidemia | TG/TC/LDL-C/HDL-C |
| Sun LX [43] | 2008 | Chinese | M/F | 30 | 53.0±8.0/54.0±6.0 | 16 weeks | 35/35 | Diabetic nephropathy | TG/TC |
| Wang FX [44] | 2015 | Chinese | M/F | 30 | 60.0±3.6/60.1±4.8 | 2 weeks | 22/28 | Diabetic nephropathy | LDL-C |
| Jin HG [45] | 2013 | Chinese | M/F | 40 | NA | 14 days | 30/30 | Coronary artery disease | LDL-C |
| Li GL [53] | 2010 | Chinese | M/F | 40 | 69.7±7.8/68.7±6.6 | 4 weeks | 102/102 | Hypertension | TC/LDL-C |
| Zhang HX [54] | 2009 | Chinese | M/F | 30 | 63.0/64.0 | 14 days | 80/80 | Coronary artery disease | TG/TC |
| Zhang C [55] | 2016 | Chinese | M/F | 30 | 46.7±12.9/47.0±12.8 | 2 weeks | 30/30 | Diabetic nephropathy | TG/TC |
| Feng K [56] | 2007 | Chinese | M/F | 40 | 67.2±16.2/65.6±17.3 | 4 weeks | 46/45 | Coronary artery disease | TG/TC/LDL-C/HDL-C |
| Qi JJ [57] | 2014 | Chinese | M/F | 10 | 64.1±5.3 | 3 months | 51/54 | Cerebrovascular disease | TG/TC/LDL-C/HDL-C |
| Lu J [58] | 2016 | Chinese | M/F | 60 | 72.5±1.5/72.4±1.4 | 1 month | 60/60 | Cerebrovascular disease | TG/TC/LDL-C/HDL-C |
| Sun JC [59] | 2016 | Chinese | M/F | 20 | 55.3±7.8 | 2 weeks | 63/63 | Hyperlipidemia | TG/TC/LDL-C/HDL-C |
| Miu L [60] | 2013 | Chinese | M/F | 30 | 59.5±2.6/58.9±2.8 | 14 days | 30/30 | Coronary artery disease | TG/TC/LDL-C/HDL-C |
| Li L [61] | 2010 | Chinese | M/F | 20 | 67.6±5.6/65.3±5.8 | 4 weeks | 45/50 | Diabetic nephropathy | TG/TC/LDL-C |
| Zhang CH [62] | 2015 | Chinese | M/F | 20 | 56.6±8.1/57.2±7.9 | 60 days | 41/41 | Cerebrovascular disease | TG/TC/LDL-C/HDL-C |
| Geng HL [63] | 2016 | Chinese | M/F | 30 | 74.3±1.4/71.3±2.3 | 14 days | 36/36 | Cerebrovascular disease | TC |

**Table S2. Characteristics of the studies included in the meta-analysis for Huangqi injection.**

| First author,  reference | Year | Ethnicity | Gender | Dose (mL) | Age (year)  control/treatment | Study duration | Numbers  Control/treatment | Study population | Outcomes |
| --- | --- | --- | --- | --- | --- | --- | --- | --- | --- |
| Zhao YJ [46] | 2015 | Chinese | M/F | 40 | 68.4±8.7/67.6±8.2 | 4 weeks | 25/25 | Hypertension | TG/TC/LDL-C/HLD-C |
| Li XY [47] | 2012 | Chinese | M/F | 40 | 56.0±7.0/55.0±8.0 | 4 weeks | 30/30 | Nephrotic syndrome | TG/TC |
| Ming H [48] | 2016 | Chinese | M/F | 20 | 60.4±9.2/61.2±8.5 | 6 months | 54/62 | Cerebrovascular disease | TG/TC/LDL-C/HLD-C |
| Wu CL [49] | 2010 | Chinese | M/F | 40 | 59.0±7.0 | 20-30 days | 40/40 | Nephrotic syndrome | TG/TC |
| Wang GJ [50] | 2009 | Chinese | M/F | 30 | 50.2±8.1/51.3±6.8 | 3 weeks | 40/40 | Nephrotic syndrome | TG/TC |
| Yu WZ [51] | 2011 | Chinese | M/F | 40 | 30.1±12.1/31.3±13.9 | 4 weeks | 27/27 | Nephrotic syndrome | TG/TC/LDL-C/HLD-C |
| Liu MH [52] | 2014 | Chinese | M/F | 1-2mL/kg/d | 6.5±2.5 | 4 weeks | 51/51 | Nephrotic syndrome | TC |

**Table S3. The lipid level changes from pre- to post-treatment with Danhong injection in control or treatment groups.**

| Reference | | Variables | Control group | | | Treatment group | | |
| --- | --- | --- | --- | --- | --- | --- | --- | --- |
|  |  |  | Pre-treatment | Post-treatment | Change | Pre-treatment | Post-treatment | Change |
| Jia QY [13] | | TG, mmol/L | 2.81±1.18 | 2.07±1.14 | -0.74±1.16 | 2.94±1.31 | 1.96±1.13 | -0.98±1.23 |
|  |  | TC, mmol/L | 7.01±0.54 | 6.85±1.03 | -0.16±0.89 | 7.14±0.49 | 6.25±0.65 | -0.89±0.59 |
|  |  | LDL-C, mmol/L | 5.14±0.35 | 4.28±0.34 | -0.86±0.35 | 4.99±0.31 | 4.06±0.37 | -0.93±0.34 |
|  |  | HDL-C, mmol/L | 1.16±0.18 | 1.41±0.12 | 0.25±0.16 | 1.24±0.27 | 1.49±0.23 | 0.25±0.25 |
| Zhang XP [14] | | TG, mmol/L | 2.93±0.97 | 2.45±0.91 | -0.48±0.94 | 2.85±0.94 | 2.01±0.88 | -0.84±0.91 |
|  |  | TC, mmol/L | 4.77±1.14 | 4.69±0.94 | -0.08±1.05 | 4.86±0.91 | 4.66±0.86 | -0.20±0.89 |
|  |  | LDL-C, mmol/L | 3.38±0.64 | 3.03±0.54 | -0.35±0.60 | 3.35±0.78 | 2.93±0.69 | -0.42±0.74 |
|  |  | HDL-C, mmol/L | 1.17±0.35 | 1.19±0.37 | 0.02±0.36 | 1.12±0.32 | 1.16±0.41 | 0.04±0.37 |
| Lu YY [15] | | TG, mmol/L | 3.04±1.10 | 2.12±1.01 | -0.92±1.06 | 3.22±1.11 | 1.75±0.71 | -1.47±0.97 |
|  |  | LDL-C, mmol/L | 5.24±1.56 | 4.07±1.41 | -1.17±1.49 | 5.67±1.66 | 3.42±1.21 | -2.25±1.49 |
| Yin XY [16] | | TG, mmol/L | 2.83±0.67 | 2.52±0.82 | -0.31±0.76 | 2.85±0.72 | 1.81±0.29 | -1.04±0.63 |
|  |  | TC, mmol/L | 8.53±0.69 | 7.64±0.78 | -0.89±0.74 | 8.32±0.58 | 5.44±0.35 | -2.88±0.51 |
| Liao SD [17] | | TG, mmol/L | 2.93±0.82 | 2.57±0.86 | -0.36±0.84 | 2.86±0.77 | 1.98±0.49 | -0.88±0.68 |
|  |  | TC, mmol/L | 6.21±1.37 | 5.89±0.96 | -0.32±1.22 | 6.18±1.24 | 5.16±1.12 | -1.02±1.18 |
|  |  | LDL-C, mmol/L | 4.45±0.64 | 3.96±1.21 | -0.49±1.05 | 4.37±0.87 | 3.23±0.58 | -1.04±0.77 |
|  |  | HDL-C, mmol/L | 1.32±0.68 | 1.45±0.71 | 0.13±0.70 | 1.27±0.63 | 1.86±0.75 | 0.59±0.70 |
| Bi ZY [18] | | TG, mmol/L | 3.43±1.14 | 3.28 ±1.05 | -0.15±1.10 | 3.41±1.11 | 2.03±0.85 | -1.38±1.01 |
|  |  | TC, mmol/L | 6.80±0.75 | 6.47±0.95 | -0.33±0.87 | 6.82±0.70 | 5.27±1.10 | -1.55±0.96 |
|  |  | LDL-C, mmol/L | 5.26±0.97 | 4.84±1.02 | -0.42±1.00 | 5.24±0.94 | 3.84±0.91 | -1.40±0.92 |
|  |  | HDL-C, mmol/L | 1.39±0.20 | 1.42±0.17 | 0.03±0.17 | 1.36±0.21 | 1.67±0.10 | 0.31±0.18 |
| Liu Y [19] | | TG, mmol/L | 2.59±0.66 | 2.22±0.68 | -0.37±0.67 | 2.63±0.87 | 184±0.51 | -0.79±0.76 |
|  |  | TC, mmol/L | 6.72±1.68 | 5.81±1.55 | -0.91±1.62 | 6.95±1.27 | 5.06±1.03 | -1.89±1.17 |
|  |  | LDL-C, mmol/L | 2.97±1.14 | 2.40±1.03 | -0.57±1.09 | 3.09±1.25 | 1.87±0.86 | -1.22±1.11 |
|  |  | HDL-C, mmol/L | 1.45±0.67 | 1.10±0.59 | -0.35±0.63 | 1.47±0.86 | 0.83±0.35 | -0.64±0.75 |
| Bi ZY [20] | | TG, mmol/L | 3.21±0.67 | 1.85±0.51 | -1.36±0.61 | 3.24±0.60 | 1.28±0.57 | -1.96±0.59 |
|  |  | TC, mmol/L | 8.53±1.12 | 6.40±1.23 | -2.13±1.18 | 8.55±1.12 | 5.64±0.98 | -2.19±1.06 |
|  |  | LDL-C, mmol/L | 4.59±0.80 | 3.40±0.65 | -1.19±0.74 | 4.61±0.80 | 2.63±0.75 | -1.98±0.78 |
|  |  | HDL-C, mmol/L | 1.24±0.26 | 1.73±0.16 | 0.49±0.23 | 1.22±0.30 | 2.17±0.30 | 0.95±0.30 |
| Zhu ZQ [21] | | TG, mmol/L | 1.90±0.80 | 1.70±0.30 | -0.20±0.70 | 1.80±0.50 | 1.20±0.40 | -0.60±0.46 |
|  |  | TC, mmol/L | 5.70±0.90 | 5.40±0.70 | -0.30±0.82 | 5.80±1.00 | 3.60±0.90 | -2.20±0.95 |
|  |  | LDL-C, mmol/L | 3.80±1.40 | 3.60±1.20 | -0.20±1.31 | 3.90±1.50 | 3.00±0.80 | -0.90±1.30 |
|  |  | HDL-C, mmol/L | 1.30±0.60 | 1.50±0.40 | 0.20±0.53 | 1.20±0.40 | 1.30±0.30 | 0.10±0.36 |
| Zhang CJ [22] | | TG, mmol/L | 2.65±0.32 | 2.41±0.27 | -0.24±0.30 | 2.61±0.36 | 1.82±0.32 | -0.79±0.34 |
|  |  | TC, mmol/L | 6.09±0.64 | 5.12±0.32 | -0.97±0.55 | 6.11±0.63 | 4.60±0.24 | -1.51±0.55 |
|  |  | LDL-C, mmol/L | 4.31±0.46 | 3.36±0.33 | -0.95±0.41 | 4.36±0.42 | 2.61±0.34 | -1.75±0.39 |
|  |  | HDL-C, mmol/L | 1.01±0.30 | 1.35±0.23 | 0.34±0.27 | 1.03±0.28 | 1.71±0.22 | 0.68±0.26 |
| Chen RH [23] | | TG, mmol/L | 2.45±0.87 | 1.95±0.56 | -0.50±0.76 | 2.47±0.95 | 1.08±0.51 | -1.39±0.82 |
|  |  | TC, mmol/L | 6.50±0.72 | 5.71±0.73 | -0.79±0.73 | 6.56±0.67 | 4.85±0.76 | -1.71±0.72 |
|  |  | LDL-C, mmol/L | 3.58±0.78 | 2.84±0.72 | -0.74±0.75 | 3.55±0.84 | 2.13±0.38 | -1.42±0.73 |
|  |  | HDL-C, mmol/L | 0.98±0.24 | 1.12±0.31 | 0.14±0.28 | 0.96±0.21 | 0.92±0.25 | -0.04±0.23 |
| Wang Y [24] | | TG, mmol/L | 3.45±1.15 | 3.29±1.06 | -0.16±1.11 | 3.42±1.12 | 2.05±0.86 | -1.37±1.02 |
|  |  | TC, mmol/L | 6.81±0.76 | 6.49±0.96 | -0.32±0.88 | 6.83±0.72 | 5.29±1.11 | -1.54±0.98 |
|  |  | LDL-C, mmol/L | 5.27±0.99 | 4.85±1.03 | -0.42±1.01 | 5.25±0.95 | 3.85±0.92 | -1.40±0.94 |
|  |  | HDL-C, mmol/L | 1.40±0.21 | 1.43±0.19 | 0.03±0.20 | 1.37±0.22 | 1.69±0.12 | 0.32±0.19 |
| Yin XH [25] | | TG, mmol/L | 1.74±0.67 | 1.68±0.56 | -0.06±0.62 | 2.39±0.68 | 2.05±0.66 | -0.34±0.67 |
|  |  | TC, mmol/L | 4.65±0.84 | 4.32±0.78 | -0.33±0.81 | 5.60±0.79 | 4.41±0.18 | -1.19±0.72 |
|  |  | LDL-C, mmol/L | 2.87±0.66 | 2.59±0.57 | -0.28±0.62 | 3.95±1.34 | 3.01±0.86 | -0.94±1.18 |
|  |  | HDL-C, mmol/L | 1.07±0.18 | 0.98±0.11 | -0.09±0.16 | 1.48±0.20 | 1.39±0.21 | -0.09±0.21 |
| Li Y [26] | | TG, mmol/L | 2.92±0.63 | 2.75±0.59 | -0.17±0.61 | 3.09±0.62 | 2.23±0.42 | -0.86±0.55 |
|  |  | TC, mmol/L | 6.20±1.23 | 5.98±1.13 | -0.22±1.18 | 6.29±1.26 | 5.34±0.98 | -0.95±1.15 |
|  |  | LDL-C, mmol/L | 4.21±0.49 | 4.06±0.51 | -0.15±0.50 | 4.22±0.52 | 3.21±0.44 | -1.01±0.48 |
|  |  | HDL-C, mmol/L | 1.38±0.24 | 1.46±0.34 | 0.08±0.30 | 1.34±0.35 | 1.89±0.41 | 0.55±0.38 |
| Jiang QF [27] | | TG, mmol/L | 1.80±0.80 | 1.60±0.30 | -0.20±0.70 | 2.00±0.50 | 1.30±0.20 | -0.70±0.44 |
|  |  | TC, mmol/L | 6.30±0.20 | 4.30±0.90 | -2.00±0.82 | 5.90±0.40 | 3.50±1.30 | -2.40±1.15 |
|  |  | LDL-C, mmol/L | 3.90±1.10 | 3.50±1.60 | -0.40±1.42 | 3.80±1.40 | 2.80±1.10 | -1.00±1.28 |
|  |  | HDL-C, mmol/L | 1.40±0.50 | 1.60±0.20 | 0.20±0.44 | 1.10±0.30 | 1.50±0.90 | 0.40±0.79 |
| Li BZ [28] | | TG, mmol/L | 2.94±1.27 | 2.74±1.18 | -0.20±1.23 | 2.86±1.41 | 2.18±1.22 | -0.67±1.33 |
|  |  | TC, mmol/L | 4.20±1.30 | 3.94±1.22 | -0.26±1.26 | 4.37±1.28 | 3.52±1.06 | -0.85±1.19 |
|  |  | LDL-C, mmol/L | 2.98±1.35 | 3.18±1.39 | -0.20±1.37 | 2.91±1.24 | 2.93±1.22 | 0.02±1.23 |
|  |  | HDL-C, mmol/L | 1.54±1.07 | 1.67±1.00 | 0.13±1.04 | 1.66±1.02 | 1.81±1.02 | 0.15±1.02 |
| Fan M [29] | | TG, mmol/L | 2.56±1.99 | 2.44±1.71 | -0.12±1.87 | 2.69±0.75 | 2.32±0.41 | -0.37±0.65 |
|  |  | TC, mmol/L | 6.28±1.72 | 6.02±2.11 | -0.26±1.94 | 6.55±1.14 | 5.21±0.89 | -1.34±1.04 |
|  |  | LDL-C, mmol/L | 3.87±1.04 | 3.61±0.97 | -0.26±1.01 | 3.79±2.01 | 2.89±1.88 | -0.90±1.95 |
|  |  | HDL-C, mmol/L | 1.09±0.99 | 1.12±0.78 | 0.03±0.90 | 0.87±0.45 | 1.14±0.76 | 0.27±0.66 |
| Liu Z [30] | | TG, mmol/L | 2.29±0.55 | 1.82±0.34 | -0.47±0.48 | 2.32±0.53 | 1.30±0.27 | -1.02±0.46 |
|  |  | TC, mmol/L | 6.19±0.87 | 5.94±0.90 | -0.25±0.89 | 6.17±0.91 | 4.55±0.76 | -1.62±0.85 |
|  |  | LDL-C, mmol/L | 3.73±0.99 | 3.35±0.86 | -0.38±0.93 | 3.70±1.01 | 2.54±0.82 | -1.16±0.93 |
|  |  | HDL-C, mmol/L | 0.91±0.12 | 1.02±0.11 | 0.11±0.12 | 0.89±0.14 | 1.15±0.13 | 0.26±0.14 |
| Shao XP [31] | | TG, mmol/L | 3.78±0.81 | 2.69±0.64 | -1.09±0.74 | 3.83±0.80 | 2.11±0.56 | -1.72±0.71 |
|  |  | TC, mmol/L | 6.52±0.97 | 5.14±0.85 | -1.38±0.92 | 6.44±0.83 | 4.09±0.80 | -2.35±0.82 |
|  |  | LDL-C, mmol/L | 3.96±0.87 | 3.03±0.78 | -0.93±0.83 | 3.99±0.91 | 2.44±0.79 | -1.55±0.86 |
|  |  | HDL-C, mmol/L | 0.84±0.37 | 0.99±0.39 | 0.15±0.38 | 0.82±0.43 | 1.33±0.36 | 0.51±0.40 |
| Liu HQ [32] | | TG, mmol/L | 2.04±0.35 | 1.98±0.66 | -0.06±0.57 | 2.23±0.45 | 1.56±0.47 | -0.67±0.46 |
|  |  | TC, mmol/L | 6.02±1.45 | 5.88±1.74 | -0.14±1.61 | 5.79±1.64 | 4.21±1.77 | -1.58±1.71 |
|  |  | LDL-C, mmol/L | 4.18±0.85 | 3.97±1.11 | -0.21±1.01 | 4.20±0.59 | 3.30±1.26 | -0.90±1.09 |
|  |  | HDL-C, mmol/L | 1.02±0.30 | 1.12±0.15 | 0.10±0.26 | 1.09±0.25 | 1.35±0.49 | 0.26±0.42 |
| Wang S [33] | | TG, mmol/L | 2.04±0.32 | 1.82±0.33 | -0.22±0.33 | 2.08±0.34 | 1.20±0.25 | -0.88±0.31 |
|  |  | TC, mmol/L | 5.76±0.78 | 5.10±0.72 | -0.66±0.75 | 5.80±0.74 | 4.04±0.74 | -1.76±0.74 |
|  |  | LDL-C, mmol/L | 3.94±0.82 | 3.02±0.82 | -0.92±0.82 | 3.88±0.74 | 2.60±0.75 | -1.28±0.75 |
|  |  | HDL-C, mmol/L | 1.12±0.35 | 1.24±0.34 | 0.12±0.35 | 1.14±0.34 | 1.54±0.42 | 0.40±0.39 |
| Hou HX [34] | | TG, mmol/L | 1.81±0.73 | 1.73±0.68 | -0.08±0.71 | 1.85±0.54 | 1.62±0.71 | -0.23±0.64 |
|  |  | TC, mmol/L | 5.58±0.91 | 5.12±0.65 | -0.46±0.81 | 5.63±0.72 | 3.12±0.73 | -2.51±0.73 |
|  |  | LDL-C, mmol/L | 3.65±0.47 | 3.76±0.74 | -0.11±0.65 | 3.22±0.37 | 3.28±0.49 | 0.06±0.44 |
|  |  | HDL-C, mmol/L | 0.82±0.18 | 0.92±0.18 | 0.10±0.18 | 0.85±0.11 | 0.93±0.29 | 0.08±0.25 |
| Wen QY [35] | | TG, mmol/L | 2.80±0.60 | 2.42±0.51 | -0.38±0.56 | 2.76±0.54 | 1.73±0.49 | -1.03±0.52 |
|  |  | TC, mmol/L | 6.77±1.25 | 6.23±1.00 | -0.54±1.15 | 6.80±1.21 | 5.11±0.90 | -1.69±1.09 |
|  |  | HDL-C, mmol/L | 0.34±0.05 | 0.56±0.05 | 0.22±0.05 | 0.33±0.04 | 0.78±0.06 | 0.45±0.05 |
| Li HY [36] | | TG, mmol/L | 2.74±1.89 | 2.01±1.37 | -0.73±1.69 | 2.81±1.63 | 1.50±0.95 | -1.31±1.42 |
|  |  | TC, mmol/L | 6.75±1.76 | 6.01±0.79 | -0.74±1.53 | 6.98±1.80 | 4.20±0.58 | -2.78±1.59 |
|  |  | LDL-C, mmol/L | 3.99±1.32 | 3.14±0.97 | -0.85±1.18 | 4.02±1.25 | 2.66±1.01 | -1.36±1.15 |
| Qiao L [37] | | TG, mmol/L | 3.36±0.38 | 2.50±0.33 | -0.86±0.36 | 3.35±0.37 | 2.11±0.32 | -1.24±0.35 |
|  |  | TC, mmol/L | 5.13±0.31 | 4.91±0.33 | -0.22±0.32 | 5.11±0.28 | 4.43±0.25 | -0.68±0.27 |
|  |  | LDL-C, mmol/L | 2.35±0.34 | 2.36±0.36 | 0.01±0.35 | 2.33±0.31 | 2.11±0.28 | -0.22±0.30 |
|  |  | HDL-C, mmol/L | 0.98±0.20 | 1.11±0.27 | 0.13±0.24 | 0.97±0.21 | 1.27±0.29 | 0.30±0.26 |
| Yang L [38] | | TG, mmol/L | 1.90±0.40 | 1.50±0.20 | -0.40±0.35 | 1.80±0.30 | 1.10±0.10 | -0.70±0.26 |
|  |  | TC, mmol/L | 5.90±1.20 | 4.80±0.50 | -1.10±1.04 | 5.80±0.90 | 3.40±0.40 | -2.40±0.78 |
|  |  | LDL-C, mmol/L | 3.70±0.50 | 3.40±0.70 | -0.30±0.62 | 3.90±1.10 | 3.00±0.80 | -0.90±0.98 |
|  |  | HDL-C, mmol/L | 1.30±0.30 | 1.80±0.40 | 0.50±0.36 | 1.20±0.40 | 1.90±0.80 | 0.70±0.69 |
| Zhou JX [39] | | TG, mmol/L | 2.27±0.85 | 2.04±0.70 | -0.23±0.79 | 2.30±0.95 | 1.72±0.92 | -0.58±0.94 |
|  |  | TC, mmol/L | 5.98±0.80 | 4.82±0.85 | -1.16±0.83 | 6.02±0.89 | 4.80±0.80 | -1.22±0.85 |
|  |  | LDL-C, mmol/L | 3.24±0.76 | 2.49±0.71 | -0.75±0.74 | 3.56±0.70 | 2.40±0.65 | -1.16±0.68 |
|  |  | HDL-C, mmol/L | 1.40±0.42 | 1.50±0.45 | 0.10±0.44 | 1.42±0.60 | 1.52±0.50 | 0.10±0.56 |
| Xiao Y [40] | | TC, mmol/L | 6.74±0.53 | 5.83±0.36 | -0.91±0.47 | 6.82±0.21 | 3.62±0.44 | -3.20±0.38 |
|  |  | LDL-C, mmol/L | 4.37±0.28 | 3.88±0.55 | -0.49±0.48 | 4.82±0.35 | 2.23±0.77 | -2.59±0.67 |
|  |  | HDL-C, mmol/L | 1.14±0.26 | 1.36±0.62 | 0.22±0.54 | 1.06±0.32 | 1.85±0.56 | 0.79±0.49 |
| Pang JZ [41] | | TC, mmol/L | 5.23±0.58 | 4.87±0.61 | -0.36±0.60 | 5.31±0.63 | 3.61±0.57 | -1.70±0.60 |
|  |  | LDL-C, mmol/L | 3.13±0.50 | 3.02±0.45 | -0.11±0.48 | 3.02±0.45 | 2.18±0.38 | -0.84±0.42 |
| Yu RC[42] | | TG, mmol/L | 3.29±1.19 | 2.28±0.82 | -1.01±1.05 | 3.67±1.18 | 2.03±0.82 | -1.64±1.05 |
|  |  | TC, mmol/L | 6.44±1.14 | 5.56±0.93 | -0.88±1.05 | 6.59±1.08 | 5.08±0.89 | -1.51±1.00 |
|  |  | LDL-C, mmol/L | 5.03±0.84 | 4.47±0.72 | -0.56±0.79 | 5.28±1.15 | 4.33±0.55 | -0.95±1.00 |
|  |  | HDL-C, mmol/L | 1.20±0.47 | 1.35±0.42 | 0.15±0.45 | 1.18±0.40 | 1.52±0.39 | 0.34±0.40 |
| Sun LX [43] | | TG, mmol/L | 2.80±1.31 | 2.86±1.25 | 0.06±1.28 | 2.76±1.61 | 1.89±1.04 | -0.87±1.41 |
|  |  | TC, mmol/L | 6.26±0.60 | 6.48±0.80 | 0.22±0.72 | 6.57±1.29 | 4.95±1.01 | -1.62±1.18 |
| Wang FX [44] | | LDL-C, mmol/L | 3.29±0.65 | 2.40±0.59 | -0.89±0.62 | 3.55±0.78 | 2.38±0.60 | -1.17±0.71 |
| Jin HG [45] | | LDL-C, mmol/L | 4.12±1.06 | 3.16±1.82 | -0.96±1.58 | 4.09±1.12 | 2.73±1.58 | -1.36±1.41 |
| Li GL [53] | | TC, mmol/L | 4.94±1.30 | 4.14±1.29 | -0.80±1.30 | 5.08±1.17 | 4.67±1.42 | -0.41±1.31 |
|  |  | LDL-C, mmol/L | 2.82±1.17 | 2.83±1.17 | 0.01±1.17 | 2.95±1.09 | 2.11±0.72 | -0.84±0.96 |
| Zhang HX [54] | | TG, mmol/L | 2.37±0.30 | 2.26±0.10 | -0.11±0.26 | 3.05±0.47 | 2.84±0.39 | -0.21±0.44 |
|  |  | TC, mmol/L | 6.47±0.23 | 5.91±0.41 | -0.56±0.36 | 6.61±0.30 | 5.08±0.49 | -1.53±0.43 |
| Zhang C [55] | | TG, mmol/L | 2.41±0.76 | 2.06±0.73 | -0.35±0.75 | 2.39±0.82 | 2.05±0.71 | -0.34±0.77 |
|  |  | TC, mmol/L | 6.38±0.74 | 5.92±0.71 | -0.46±0.73 | 6.31±0.77 | 4.76±0.65 | -1.55±0.72 |
| Feng K [56] | | TG, mmol/L | 1.35±0.56 | 1.30±0.47 | -0.05±0.52 | 1.48±0.61 | 1.49±0.57 | 0.01±0.59 |
|  |  | TC, mmol/L | 4.76±0.73 | 4.65±0.71 | -0.11±0.72 | 4.80±0.69 | 4.71±0.72 | -0.09±0.71 |
|  |  | LDL-C, mmol/L | 2.88±0.71 | 2.24±0.69 | -0.64±0.70 | 2.79±0.69 | 2.26±0.74 | -0.53±0.72 |
|  |  | HDL-C, mmol/L | 1.44±0.62 | 1.52±0.59 | 0.08±0.61 | 1.31±0.62 | 1.29±0.59 | -0.02±0.61 |
| Qi JJ [57] | | TG, mmol/L | 2.69±0.55 | 2.07±0.49 | -0.62±0.52 | 2.82±0.47 | 1.84±0.45 | -0.98±0.46 |
|  |  | TC, mmol/L | 6.28±0.97 | 5.65±0.90 | -0.63±0.94 | 6.42±1.15 | 5.17±0.95 | -1.25±1.06 |
|  |  | LDL-C, mmol/L | 3.24±0.75 | 2.59±0.48 | -0.65±0.66 | 3.33±0.83 | 2.39±0.54 | -0.94±0.73 |
|  |  | HDL-C, mmol/L | 1.39±0.48 | 1.61±0.42 | 0.22±0.45 | 1.33±0.55 | 1.74±0.45 | 0.41±0.51 |
| Lu J [58] | TG, mmol/L | 2.01±0.44 | 1.90±0.31 | -0.11±0.39 | 1.99±0.47 | 1.20±0.15 | -0.79±0.42 |  |
|  | TC, mmol/L | 6.22±0.74 | 5.91±0.80 | -0.31±0.77 | 6.28±0.77 | 3.20±0.41 | -3.08±0.67 |  |
|  | LDL-C, mmol/L | 4.27±0.80 | 4.02±0.61 | -0.25±0.72 | 4.31±0.81 | 3.38±0.50 | -0.93±0.71 |  |
|  | HDL-C, mmol/L | 1.33±0.19 | 1.30±0.11 | -0.03±0.17 | 1.29±0.17 | 1.82±0.13 | 0.53±0.15 |  |
| Sun JC [59] | TG, mmol/L | 2.00±0.50 | 1.60±0.40 | -0.40±0.46 | 1.90±0.70 | 1.30±0.60 | -0.60±0.66 |  |
|  | TC, mmol/L | 5.80±0.40 | 4.30±0.80 | -1.50±0.69 | 6.00±0.70 | 3.50±0.90 | -2.50±0.82 |  |
|  | LDL-C, mmol/L | 3.90±1.10 | 3.50±0.90 | -0.40±1.01 | 3.80±1.20 | 2.80±0.80 | -1.00±1.06 |  |
|  | HDL-C, mmol/L | 1.30±0.40 | 1.50±0.50 | 0.20±0.46 | 1.20±0.60 | 1.60±0.50 | 0.40±0.56 |  |
| Miu L [60] | TG, mmol/L | 2.94±0.58 | 1.59±0.72 | -1.35±0.66 | 3.12±0.46 | 1.82±0.58 | -1.30±0.53 |  |
|  | TC, mmol/L | 5.98±0.96 | 4.02±0.65 | -1.96±0.85 | 6.24±0.87 | 3.65±0.74 | -2.59±0.81 |  |
|  | LDL-C, mmol/L | 3.87±0.51 | 2.01±0.64 | -1.86±0.59 | 4.08±0.46 | 1.73±0.52 | -2.35±0.49 |  |
|  | HDL-C, mmol/L | 1.12±0.46 | 1.28±0.56 | 0.16±0.52 | 1.09±0.54 | 1.58±0.45 | 0.49±0.50 |  |
| Li L [61] | TG, mmol/L | 2.69 ±0.85 | 2.56±0.77 | -0.13±0.81 | 2.72±0.82 | 1.75±0.41 | -0.97±0.71 |  |
|  | TC, mmol/L | 6.09 ±1.26 | 5.67±1.24 | -0.42±1.25 | 6.12±1.22 | 4.23±0.76 | -1.89±1.07 |  |
|  | LDL-C, mmol/L | 3.84 ±0.45 | 3.53±0.41 | -0.31±0.43 | 3.86±0.44 | 3.01±0.43 | -0.85±0.44 |  |
| Zhang CH [62] | TG, mmol/L | 1.90±0.40 | 1.50±0.30 | -0.40±0.36 | 1.80±0.40 | 1.10±0.10 | -0.70±0.36 |  |
|  | TC, mmol/L | 5.90±1.00 | 4.80±0.60 | -1.10±0.87 | 5.80±0.80 | 3.30±0.40 | -2.50±0.69 |  |
|  | LDL-C, mmol/L | 3.70±0.80 | 3.40±0.80 | -0.30±0.80 | 3.80±1.10 | 2.90±0.70 | -0.90±0.96 |  |
|  | HDL-C, mmol/L | 1.20±0.30 | 1.60±0.40 | 0.40±0.36 | 1.30±0.40 | 1.90±0.80 | 0.60±0.69 |  |
| Geng HL [63] | TC, mmol/L | 5.91±0.90 | 5.40±0.73 | -0.51±0.83 | 5.97±0.92 | 4.74±0.61 | -1.23±0.81 |  |

**Table 4. The lipid level changes from pre- to post-treatment with Huangqi injection in control or treatment groups.**

| Reference | Variables | Control group | | | Treatment group | | |
| --- | --- | --- | --- | --- | --- | --- | --- |
|  |  | Pre-treatment | Post-treatment | Change | Pre-treatment | Post-treatment | Change |
| Zhao YJ [46] | TG, mmol/L | 1.56±0.10 | 1.52±0.08 | -0.04±0.09 | 1.54±0.10 | 1.47±0.09 | -0.07±0.10 |
|  | TC, mmol/L | 5.22±0.56 | 4.96±0.54 | -0.26±0.55 | 5.23±0.61 | 4.69±0.62 | -0.54±0.62 |
|  | LDL-C, mmol/L | 3.01±0.11 | 2.97±0.11 | -0.04±2.92 | 3.02±0.12 | 2.95±0.12 | -0.07±2.89 |
|  | HDL-C, mmol/L | 1.51±0.16 | 1.53±0.15 | -0.02±0.55 | 1.52±0.25 | 1.58±0.23 | -0.06±0.24 |
| Li XY [47] | TG, mmol/L | 3.62±0.86 | 3.23±0.67 | -0.39±0.78 | 3.58±0.82 | 2.31±0.42 | -1.27±0.71 |
|  | TC, mmol/L | 8.96±1.38 | 8.64±1.15 | -0.32±1.28 | 8.98±1.46 | 6.72±1.21 | -2.26±1.35 |
| Ming H [48] | TG, mmol/L | 2.71±0.42 | 1.95±0.27 | -0.76±0.37 | 2.66±0.36 | 1.41±0.15 | -1.25±0.31 |
|  | TC, mmol/L | 6.24±1.25 | 5.81±0.88 | -0.43±1.11 | 6.17±1.38 | 5.16±0.79 | -1.01±1.20 |
|  | LDL-C, mmol/L | 3.62±0.41 | 2.70±0.28 | -0.92±2.52 | 3.57±0.35 | 2.36±0.23 | -1.21±2.21 |
|  | HDL-C, mmol/L | 1.04±0.16 | 1.10±0.18 | -0.06±0.17 | 1.08±0.14 | 1.13±0.21 | -0.05±0.19 |
| Wu CL [49] | TG, mmol/L | 1.90±0.40 | 1.30±0.50 | -0.60±0.46 | 1.80±0.60 | 1.70±0.40 | -0.10±0.53 |
|  | TC, mmol/L | 6.10±1.30 | 4.60±0.80 | -1.50±1.14 | 6.00±1.60 | 5.80±0.40 | -0.20±1.44 |
| Wang GJ [50] | TG, mmol/L | 2.87±0.16 | 2.84±0.18 | -0.03±0.17 | 2.85±0.15 | 1.87±0.19 | -0.98±0.17 |
|  | TC, mmol/L | 5.35±0.45 | 5.28±0.35 | -0.07±0.41 | 5.22±0.40 | 3.25±0.26 | -1.97±0.35 |
| Yu WZ [51] | TG, mmol/L | 3.16±1.19 | 1.98±0.68 | -1.18±1.03 | 3.14±1.29 | 1.56±0.46 | -1.58±1.13 |
|  | TC, mmol/L | 10.75±2.59 | 8.22±2.37 | -2.53±2.49 | 10.91±2.61 | 6.75±1.64 | -4.16±2.29 |
|  | LDL-C, mmol/L | 7.59±4.51 | 5.31±2.89 | -2.28±4.96 | 7.48±3.15 | 3.81±2.24 | -3.67±3.53 |
|  | HDL-C, mmol/L | 0.95±0.10 | 1.35±0.12 | -0.40±0.11 | 0.96±0.11 | 1.45±0.13 | -0.49±0.12 |
| Liu MH [52] | TC, mmol/L | 9.26±2.27 | 4.99±1.28 | -4.27±1.97 | 9.35±2.16 | 2.99±1.28 | -6.36±1.88 |
