# Supplementary material for: The lipid-lowering effects of Danhong and Huangqi injections: a meta-analysis of clinical controlled trials
Source: Lipids Health Dis. 2018 May 10;17:106. doi: 10.1186/s12944-018-0760-2 (PMC5944065; doi:10.1186/s12944-018-0760-2)
Supplement: Supplementary file 2 — Figures S1-S4. Galbraith plots of the association analysis between Danhong injection and the changes in plasma levels of TG, TC, LDL-C and HDL-C, respectively; Figures S5-S7. Galbraith plots of the association analysis between Huangqi injection and the changes in plasma levels of TG, TC and HDL-C, respectively; Figures S8-S11. Begg’s funnel plots of the association analysis between Danhong injection and the changes in plasma levels of TG, TC, LDL-C and HDL-C, respectively; Figures S12-S15. Begg’s funnel plots of the association analysis between Huangqi injection and the changes in plasma levels of TG, TC, LDL-C and HDL-C, respectively. (DOCX 54 kb) [file 12944_2018_760_MOESM2_ESM.docx]

**Supplemental Figures:**

**Figure S1.** Galbraith plot of the association analysis between Danhong injection and the change in TG levels.

**Figure S2.** Galbraith plot of the association analysis between Danhong injection and the change in TC levels.

**Figure S3.** Galbraith plot of the association analysis between Danhong injection and the change in LDL-C levels.

**Figure S4.** Galbraith plot of the association analysis between Danhong injection and the change in HDL-C levels.

**Figure S5.** Galbraith plot of the association analysis between Huangqi injection and the change in TG levels.

**Figure S6.** Galbraith plot of the association analysis between Huangqi injection and the change in TC levels.

**Figure S7.** Galbraith plot of the association analysis between Huangqi injection and the change in HDL-C levels.

**Figure S8.** Begg’s funnel plot of the association analysis between Danhong injection and the change in TG levels.

**Figure S9.** Begg’s funnel plot of the association analysis between Danhong injection and the change in TC levels.

**Figure S10.** Begg’s funnel plot of the association analysis between Danhong injection and the change in LDL-C levels.

**Figure S11.** Begg’s funnel plot of the association analysis between Danhong injection and the change in HDL-C levels.

**Figure S12.** Begg’s funnel plot of the association analysis between Huangqi injection and the change in TG levels.

**Figure S13.** Begg’s funnel plot of the association analysis between Huangqi injection and the change in TC levels.

**Figure S14.** Begg’s funnel plot of the association analysis between Huangqi injection and the change in LDL-C levels.

**Figure S15.** Begg’s funnel plot of the association analysis between Huangqi injection and the change in HDL-C levels.

**Figure S1.** Galbraith plot of the association analysis between Danhong injection and the change in TG levels.

**Figure S2.** Galbraith plot of the association analysis between Danhong injection and the change in TC levels.

**Figure S3.** Galbraith plot of the association analysis between Danhong injection and the change in LDL-C levels.

**Figure S4.** Galbraith plot of the association analysis between Danhong injection and the change in HDL-C levels.

**Figure S5.** Galbraith plot of the association analysis between Huangqi injection and the change in TG levels.

**Figure S6.** Galbraith plot of the association analysis between Huangqi injection and the change in TC levels.

**Figure S7.** Galbraith plot of the association analysis between Huangqi injection and the change in HDL-C levels.

**Figure S8.** Begg’s funnel plot of the association analysis between Danhong injection and the change in TG levels.

**Figure S9.** Begg’s funnel plot of the association analysis between Danhong injection and the change in TC levels.

**Figure S10.** Begg’s funnel plot of the association analysis between Danhong injection and the change in LDL-C levels.

**Figure S11.** Begg’s funnel plot of the association analysis between Danhong injection and the change in HDL-C levels.

**Figure S12.** Begg’s funnel plot of the association analysis between Huangqi injection and the change in TG levels.

**Figure S13.** Begg’s funnel plot of the association analysis between Huangqi injection and the change in TC levels.

**Figure S14.** Begg’s funnel plot of the association analysis between Huangqi injection and the change in LDL-C levels.

**Figure S15.** Begg’s funnel plot of the association analysis between Huangqi injection and the change in HDL-C levels.
